# Rapid Detection of Tomato Spotted Wilt Virus With Cas13a in Tomato and *Frankliniella occidentalis*

**DOI:** 10.3389/fmicb.2021.745173

**Published:** 2021-10-20

**Authors:** Wanhong Zhang, Yubing Jiao, Chengying Ding, Lili Shen, Ying Li, Yanbi Yu, Kun Huang, Bin Li, Fenglong Wang, Jinguang Yang

**Affiliations:** ^1^Key Laboratory of Tobacco Pest Monitoring Controlling and Integrated Management, Tobacco Research Institute of Chinese Academy of Agricultural Sciences, Qingdao, China; ^2^Country Yunnan Province Company of China Tobacco Corporation, Kunming, China; ^3^Honghe City Company of Yunnan Tobacco Company, Mile, China; ^4^Sichuan Province Company of China Tobacco Corporation, Chengdu, China

**Keywords:** CRISPR-cas13a, RPA, virus detection, tomato spotted wilt virus, *Frankliniella occidentalis*

## Abstract

As one of the top 10 plant viruses, the severity of losses to crop productivity caused by the tomato spotted wilt virus (TSWV) has resulted in an urgent need to develop a more sensitive and rapid method of detection. In this study, we developed a CRISPR/Cas13a-based detection system to diagnose TSWV in tomato and western flower thrips (*Frankliniella occidentalis*). The detection system relies on recombinase polymerase amplification and Cas13a-mediated collateral cleavage activity. Positive results can be distinguished after 20 min by a significantly enhanced fluorescence signal. We tested the sensitivity of CRISPR/Cas13a-based detection system and found that the detection system that we developed has limits of detection that reaches 2.26 × 10^2^ copies/μl and a 10-fold increase compared with the sensitivity of using RT-PCR to detect the virus. Furthermore, the CRISPR/Cas13a-based detection system has a high selectivity for the TSWV without interference from other viruses. The CRISPR/Cas13a-based detection system was utilized to detect the TSWV in samples of tomato leaves and the transmission vector *F. occidentalis* that were fully consistent with the results when RT-PCR was used to detect the virus.

## Graphical Abstract

Schematic of CRISPR/Cas13-based virus detection.

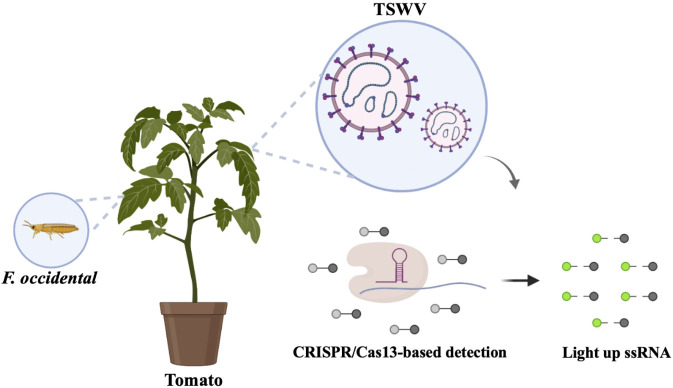

## Introduction

Plant diseases caused by infection with tospoviruses have been causing enormous crop losses worldwide (Oliver and Whitfield, 2016). The tomato spotted wilt virus (TSWV), the type species of the genus *Orthotospovirus*, the family *Tospoviridae*, and the order *Bunyavirales*, is a tripartite negative/ambisense ssRNA virus. Based on their molecular weight, the three ssRNAs have been designated RNA-S, RNA-L, and RNA-M. RNA-L is the negative sense RNA, which encodes RNA-dependent RNA polymerase (RdRp). RNA-M and RNA-S are ambisense RNAs, which encode the movement protein NSm and the glycoprotein Gn/Gc, respectively. RNA-S encodes the silencing suppressor NSs and the nucleocapsid protein N. The TSWV has a worldwide distribution and infects more than 1,000 species of plants in more than 80 families (Pappu et al., 2009). The TSWV is transmitted in a persistent propagative manner by western flower thrips. Thrips acquire the virus during larval stages, and only thrips adults that acquired the virus as larvae can transmit the pathogen (Oliver and Whitfield, 2016). The characteristic symptom of the TSWV is the appearance of obvious concentric rings on the leaves and fruits, which later turn brown. The leaves may appear chlorotic and bronze, and premature senescence starts from the old leaves (Wangai et al., 2001). Long-term infection can cause the death of the entire plant, posing a grave threat to the planting and harvesting of crops (Turina et al., 2016; Yu et al., 2020). It is critical to establish a more efficient and sensitive method to detect the TSWV during a plant virus quarantine. The occurrence of viral diseases can be predicted in the early stages, and reasonable prevention and control measures can be taken to prevent the outbreak of these diseases in a timely manner. Current strategies in plant virus diagnosis primarily include nucleic acid-based detection methods and antigen-based detection tests (Boonham et al., 2002, 2014; Tantiwanich et al., 2018). The existing nucleic acid detection methods for plant viruses are sensitive and rapid, but they require expensive instruments and cannot be extensively applied in the field. Although the method to detect common viruses using antigen–antibody reactions can be conducted without expensive instruments, a substantial amount of time and money is consumed during the process of establishing a method to test new viruses (Khater et al., 2017). In addition to traditional methods, isothermal amplification techniques, such as recombinase polymerase amplification (RPA) and loop-mediated isothermal amplification (LAMP), are also increasingly used to detect viruses (Sui et al., 2018; Lee et al., 2021). The isothermal amplification technology does not require expensive equipment, but when agarose gel electrophoresis is used to verify the isothermal amplification reactions, the lower visual resolution and the contamination caused by aerosol DNA during electrophoresis will affect the sensitivity and specificity of the detection results (Babu et al., 2018).

Recently, the clustered regularly interspaced short palindromic repeats (CRISPR) and CRISPR-associated (Cas) systems have been used as important gene editing tools to perform studies related to viral diagnostics (Myhrvold et al., 2018; Hadidi, 2019). In the CRISPR/Cas systems, a single CRISPR RNA (crRNA) guides a single protein effector (Cas protein) to target, cleave, and edit the specific nucleotide (Abudayyeh et al., 2016). The unique RNA-targeting mechanism of CRISPR/Cas13a systems provides an ability to detect viruses. The Cas13a-based SHERLOCK (Specific High Sensitivity Enzymatic Reporter Unlocking) platform was developed by the Zhang group to detect the Zika (ZIKV) and dengue (DENV) viruses, and the high sensitivity of this protocol was used to identify region-specific strains of the viruses (Gootenberg et al., 2017; Kellner et al., 2019). The SHERLOCK system uses RPA to amplify a piece of nucleic acid that contains the target sequence of the CRISPR system. The CRISPR system cleaves the probe with a fluorescent group and a quencher group at both ends, and the fluorescent signal generated in a short time will distinguish the positive samples. CRISPR/Cas-based diagnostic systems, such as Cas9, Cas12, Cas13, and Cas14, have been used to detect nucleic acids. Among them, Cas9, Cas12, and Cas14 target ssDNA, so they are primarily used for DNA viruses, such as the African swine fever virus (ASFV)(Wang et al., 2020) and the SARS-CoV-2 (Broughton et al., 2020). The HOLMESv2 established by the Wang group has four different detection functions, and the system uses Cas12 for critical work in molecular diagnoses and epigenetics, such as single-nucleotide polymorphism (SNP), mRNA, circular RNA, and the degree of DNA methylation (Li et al., 2019). Cas13 targets ssRNA and can be used to detect RNA viruses, such as the avian influenza A (H7N9)(Liu et al., 2019) and the Ebola virus (Qin et al., 2019).

Currently, CRISPR/Cas12-based plant viral detection methods have been established. An example is the detection of common apple viruses combined with gold nanoparticles to visualize the results (Jiao et al., 2021). Other examples include Cas12-based tomato-related virus detection (Alon et al., 2021), and the detection of potexvirus, potyvirus, tobamovirus, and other types of plant RNA viruses (Aman et al., 2020), but the CRISPR technique is still rarely utilized to detect plant viruses. In this study, we developed a method to detect the TSWV based on the CRISPR/Cas13a system. This assay is performed in three steps. First, a cDNA of the plant sample to be tested is obtained. Second, the sample cDNA is used as a template for the RPA reaction, which takes 30 min at 39°C. Finally, the product of the RPA reaction is added to the CRISPR-based detection system, and a fluorescence signal that occurs after 15 min at 37°C determines the presence of the TSWV in the sample. The new assay that combines RPA with Cas13a-mediated collateral cleavage activity is highly sensitive compared with RPA or RT-PCR and highly specific for the TSWV. In addition, the minimum concentration of the selected TSWV target sequence that can be detected is 2.26 × 10^2^ copies/μl. Furthermore, the method was applied to detect the TSWV in samples of tomato leaves and the transmission vector *F. occidentalis*, thus, demonstrating that it is a strong candidate for the detection of plant viruses.

## Materials and Methods

### Materials

The TSWV (GenBank: MN861978.1), tomato zonate spot virus (TZSV) (GenBank: MG656995.1), tomato mosaic virus (TMV) (GenBank: HE818419), cucumber mosaic virus (CMV) (GenBank: MG190364.1), and potato virus Y (PVY) (GenBank: HM590405.1) have all been isolated and preserved in our laboratory. The tomato samples and *F. occidentalis* were collected from a field of tomatoes in Mile City, Sichuan Province. We collected 20 tomato samples with typical symptoms of the TSWV and 20 surrounding asymptomatic tomatoes. The specimens of *F. occidentalis* were detected as non-viruliferous vectors by an RT-PCR assay that uses specific primers for the TSWV (Supplementary Table 1), and they were maintained on healthy kidney bean pods at 26°C, 70% relative humidity, and a 16-h:8-h light:dark photoperiod. The larval thrips (L1 <8 h) were picked and fed on tomatoes that were infected with the TSWV for 48 h. They were then fed with healthy beans until they became adults. The total RNA of leaves was extracted using the TRIzol reagent (Vazyme, Nanjing, China), followed by cDNA synthesis using a HiScript III RT SuperMix for qPCR (Vazyme). The total RNA of individual adult thrips was extracted with an EASYspin Plus Tissue/Cell RNA Rapid Extraction Kit (Aidlab, Beijing, China) and followed by cDNA synthesis using a HiScript III RT SuperMix for qPCR (Vazyme). The in-fusion method was used to connect the TSWV *N* gene fragment to the Fu28 vector.

### Recombinase Polymerase Amplification Primer Pesign and Preparation of CRISPR RNA

The sequences of 25 TSWV isolates were downloaded from NCBI and compared by MEGAX, and the conserved sequence was selected as the target sequence of crRNA (Supplementary Figure 4). The RPA primers were designed from the conserved nucleotide region of the TSWV *N* gene. The T7 promoter sequence was appended to the 5′ end of RPA forward primer (Supplementary Table 2). The spacer sequence of crRNA was designed to recognize the region between the RPA primer sequences. The spacer sequence was aligned using NCBI BLAST to ensure the specificity of the crRNA target sequence (Supplementary Table 3). To prepare the crRNA, a crRNA DNA template with a T7 promoter sequence was annealed to a T7 promoter sequence at a final concentration of 10 mM in annealing buffer for DNA Oligos (5 ×) (Beyotime Biotechnology, Shanghai, China) (Supplementary Table 4). The annealing reaction was conducted by denaturation at 95°C for 2 min and then a cool down by 0.1°C every 8 s to 25°C. The crRNA was transcribed using an *in vitro* Transcription T7 Kit (TaKaRa, Dalian, China). The transcription reaction was incubated for 2 h at 42°C, and the DNA in the reaction was removed using RNase-free DNase I (TaKaRa). The transcripts were purified using an RNA Clean Kit (Tiangen, Beijing, China). The crRNA was stored at −80°C.

### Purification of the LwCas13a Protein

His6-Twinstrep-SUMO-huLwCas13a (addgene: #90097) bacterial expression vectors were transformed into *Escherichia coli* Transetta (DE3) chemically competent cells (Transgen, Beijing, China). The positive colonies were inoculated in LB growth media that contained 50 μg/ml ampicillin and shaken overnight at 37°C. A volume of 4 ml of the overnight culture was added to 400 ml of LB that contained 50 μg/ml of ampicillin, and it was incubated at 37°C until the OD_600_ increased to 0.6. His6-SUMO-LwCas13a expression was induced by the addition of 0.5 mM IPTG at 16°C for 18 h. The culture was collected by centrifugation at 15,000 × *g* for 1 min at 4°C. The supernatant was removed, and the cell pellet was stored at 20°C for subsequent purification. The protein was purified using a His-tag Protein Purification Kit (Beyotime Biotechnology). The cell pellet was resuspended in non-denatured lysis buffer (50 mM Tris and 500 mM NaCl, pH 7.5) supplemented with 1 mM PMSF (Solarbio, Beijing, China) and 1 mg/ml of lysozyme and incubated on ice for 30 min. The cell pellet was sonicated on ice five times with 10 pulses of 2 s each until the liquid was clear. The lysate was centrifuged at 10,000 × *g* for 20 min in 4°C, and the supernatant was transferred into a purification column that contained 1 ml of BeyoGold^TM^ His-tag purification resin. The flow-through was passed through the column two to three times and collected for SDS-PAGE analysis. The His-tag purification resin was washed five times with 1 ml of non-denatured washing buffer (50 mM Tris, 500 mM NaCl, and 10 mM imidazole, pH 7.5) each time, and the non-denatured washing buffer was collected for analysis by 8% SDS-PAGE. The protein was eluted with non-denaturing elution buffer (50 mM Tris, 500 mM NaCl, and 250 mM imidazole, pH 7.5), and the eluted fractions were analyzed by 8% SDS-PAGE to test for the presence of His6-SUMO-LwCas13a. Every milliliter of non-denatured elution buffer that contained Cas13 protein was digested by 2 μl SUMO protease (Beyotime Biotechnology) at 4°C overnight and purified by a column that contained BeyoGold^TM^ His-tag purification resin. The Cas13a protein eluted in the non-denatured washing buffer and concentrated *via* a millipore concentrator (50,000 NMWL). The protein concentration was measured by a BCA assay and stored at −80°C.

### Clustered Regularly Interspaced Short Palindromic Repeats/Clustered Regularly Interspaced Short Palindromic Repeat-Associated 13a-Mediated Assay

The detection of the TSWV relies on RPA to amplify a segment of the *N* gene of the virus, and the amplified sequences were targeted using CRISPR/Cas13a-based detection. The transcription of T7 *in vitro* transforms the RPA amplification products to single-stranded RNA, and the RNA probe generates a significantly enhanced fluorescence signal using the LwCas13a-mediated collateral cleavage activity. Each Cas13a-based detection system contained RPA products, T7 *in vitro* transcription and the Cas13a detection reaction. The RPA reaction (TwistAmp^®^ Basic kit, TwistDX, United Kingdom) was incubated at 39°C in a thermostatic water bath for 30 min. The Cas13a detection system that contained T7 *in vitro* transcription included 100 μl of detection buffer (20 mmol/L HEPES, 60 mmol/L NaCl, and 6 mmol/L MgCl_2_, pH 6.8), 0.5 μl of 50 U/μl T7 RNA polymerase (TaKaRa), 1 μl of 50 mM ATP, 1 μl of 50 mM GTP, 1 μl of 50 mM CTP, 1 μl of 50 mM UTP (TaKaRa), 0.5 μl of 40 U/μl of recombinant RNase inhibitor (TaKaRa), 0.5 μl of purified LwCas13a (57.8 nM stock concentration), 0.5 μl of crRNA (10 ng/μl stock concentration), and 0.5 μl 20 of μM RNA reporter labeled using 5′ FAM fluorophore and 3′ BHQ1 (FAM-TUUUUUC-BHQ1) (Sangon Biotech). Each Cas13a-based detection system that contained 2 μl of RPA amplification products was conducted at 37°C, and the fluorescence values were monitored using a Tecan Infinite 200Pro plate reader (Tecan, Männedorf, Switzerland) under a 485-nm excitation wavelength and a 529-nm emission wavelength with a fluorescence intensity reading every 30 s for 45 fluorescence intensity kinetics cycles. Moreover, to determine the necessity of the detection system components without affecting the change in the fluorescence signal, we tested whether the detection system will produce changes in the fluorescence signal in the absence of components.

### Sensitivity and Specificity of the Tomato Spotted Wilt Virus Clustered Regularly Interspaced Short Palindromic Repeats/Clustered Regularly Interspaced Short Palindromic Repeat-Associated 13a-Based Detection System

The plasmid that contained the full-length fragment of the N gene at a concentration of 100 ng/μl as the original concentration had a copy number of 2.26 × 10^8^ copies/μl based on the copy number formula [copy number = 6.02 × 10^23^ × (100 ng/ul × 10^–9^)/DNA length × 660), and it was diluted to 2.26 × 10^1^copies/μl in a 10-fold gradient. The sensitivity of CRISPR/Cas13a-based detection system was determined using different concentrations of plasmid templates of RPA reactions, 0.5 μl T7 RNA polymerase, 0.5 μl of 50 U/μl T7 RNA polymerase, 1 μl 50 mM ATP, 1 μl 50 mM GTP, 1 μl 50 mM CTP, 1 μl 50 mM UTP, 0.5 μl of 40 U/μl recombinant RNase inhibitor, 0.5 μl purified LwCas13a (57.8 nM stock concentration), 0.5 μl crRNA (10 ng/μl stock concentration), and 0.5 μL 20 μM RNA reporter in 100 μl of detection buffer (20 mmol/L HEPES, 60 mmol/L NaCl, and 6 mmol/L MgCl_2_, pH 6.8). Moreover, to compare the limit of detection (LOD) with CRISPR/Cas13a-based detection, different concentrations of plasmid templates were also amplified by PCR with RPA primers. In addition, we directly purified the RPA product with a SanPrep Column PCR Product Purification Kit (Sangon) to agarose gel electrophoresis. The specificity of the CRISPR/Cas13a-based detection system was tested using TZSV, TMV, CMV, and PVY, and amplified using TSWV primers, followed by CRISPR/Cas13a-based detection. RNA was extracted from tomatoes infected with TZSV, TMV, CMV, and PVY, and HiScript III RT SuperMix for qPCR (Vazyme) was used to obtain the cDNA as template for the RPA reaction. The TSWV used plasmid as template, and the other viruses used cDNA as templates and RT-PCR has been used to detect that the source of the virus is infected (Supplementary Figure 3). All the detection reactions had three biological replicates and were conducted at 37°C, and the microplate reader was set for 45 fluorescence intensity kinetics measurements with an interval of 30 s between each measurement.

### Detection of the Tomato Spotted Wilt Virus in Plant and *Frankliniella occidentalis* Samples Using the Clustered Regularly Interspaced Short Palindromic Repeats/Cas13a-Based Detection System

To test the CRISPR/Cas13a-based detection system, A total of 40 tomato samples and 30 thrips samples were tested. The total RNA was used in HiScript III RT SuperMix for qPCR to synthesize the cDNA. The RPA reaction was incubated at 39°C for 30 min, and 2 μl of the RPA product was used for the CRISPR/Cas13a-based detection as described above. Moreover, to verify the accuracy of the CRISPR/Cas13a-based detection system, the samples were tested using RT-PCR and analyzed by polyacrylamide gel electrophoresis.

## Results

### Expression and Purification of LwCas13a

Expression of the His6-SUMO-LwCas13a protein was induced for 16 h at 18°C using 500 μM IPTG. To purify the LwCas13a protein, the 6 × His tag at the N-terminus of LwCas13a helped the protein to bind to the purification resin, and it was finally collected using non-denatured elution buffer. An SDS-PAGE analysis of the purified LwCas13a protein is shown in Supplementary Figure 1A. The His6-SUMO-LwCas13a protein is 150 KDa, and the protein was primarily present in the collection buffer and at very low levels in the flow-through and washing buffers. To maximize the RNase activity of the Cas13a protein, we used SUMO protease to remove the SUMO tag, and the LwCas13a protein was 138 KDa as shown in Supplementary Figure 1B.

### Validation of the Tomato Spotted Wilt Virus Clustered Regularly Interspaced Short Palindromic Repeats/Cas13a-Based Detection System

**FIGURE 1:**
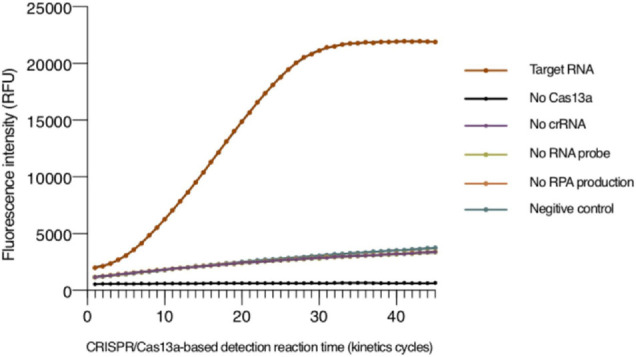
Validation of the the tomato spotted wilt virus (TSWV) clustered regularly interspaced short palindromic repeats (CRISPR)/clustered regularly interspaced short palindromic repeat-associated 13a (Cas13a)-based detection system. The positive sample in a 100-μl detection buffer contained a plasmid of the TSWV *N* gene as the template for RPA reaction, T7 RNA polymerase, NTP, recombinant RNase inhibitor, purified LwCas13a, crRNA, and RNA reporter. The detection systems were monitored in the absence of LwCas13a, crRNA, RNA probe, or RPA amplification products. The negative control utilized RNase-free water as a template of the RPA reaction. RPA, recombinant polymerase amplification.

Since the detection method that we established is based on a fluorescent signal generated in the system within some period of time, we needed to ensure that the components in the system will not affect the fluorescent signal and are necessary for the system. The result is shown in Figure 1, and the fluorescence signal was almost unchanged when the detection system lacked any LwCas13a, correct crRNA, or RPA amplification products. In contrast, the Cas13 protein will only perform its collateral cleavage activity, and the fluorescence signal only significantly changed when LwCas13a, correct crRNA, and the RPA amplification products were all present.

### Sensitivity Analysis of the Tomato Spotted Wilt Virus Clustered Regularly Interspaced Short Palindromic Repeats/Clustered Regularly Interspaced Short Palindromic Repeat-Associated 13a-Based Detection

**FIGURE 2:**
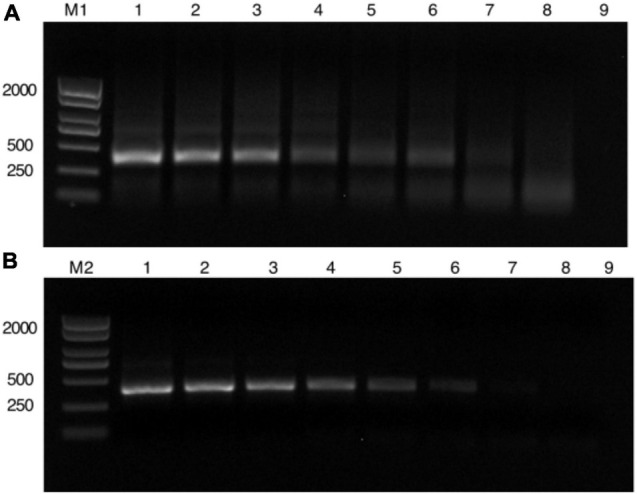
Length of detection of PCR and RPA to detect different concentrations. **(A)** Different concentrations amplified by RPA. (M1) DL2000 Plus DNA Marker. (1–8) The plasmid with *N* segment of the TSWV were serially diluted 10-fold (2.26 × 10^8^ to 2.26 × 10^1^ copies/μl). (9) Negative control has RNase-free water as an input. **(B)** Different concentrations only amplified by the PCR reaction. (M2) DL2000 Plus DNA Marker. (1–8) The plasmid with *N* segment of the TSWV were serially diluted 10-fold (2.26 × 10^8^ to 2.26 × 10^1^ copies/μl). (9) Negative control has RNase-free water as an input. RPA, recombinant polymerase amplification.

**FIGURE 3:**
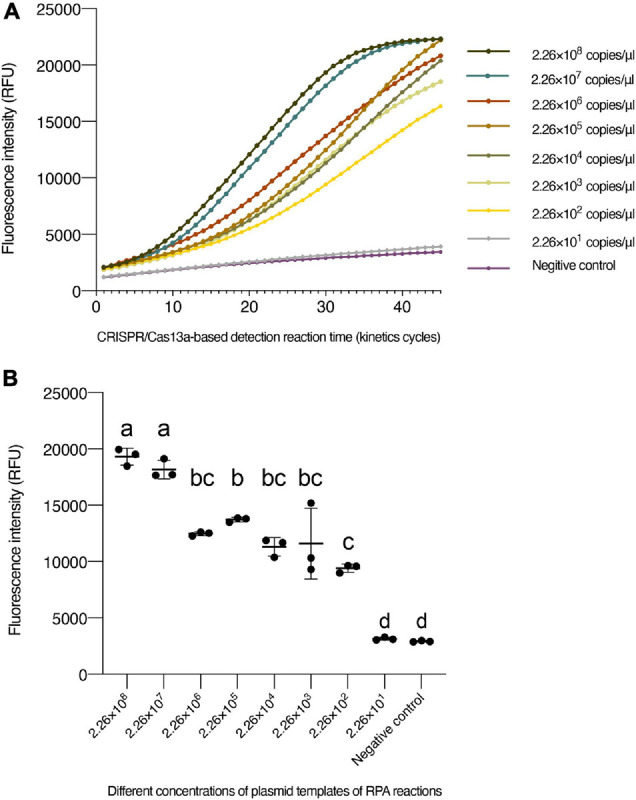
The sensitivity of CRISPR/Cas13a-based detection. **(A)** Fluorescence intensity for plasmid templates of RPA reactions with different concentrations. **(B)** Intensity of fluorescence signals generated after 15 min (30 kinetics cycles) of CRISPR/Cas13a-based detection at each dilution. The negative control has RNase-free water as an input instead of RPA production. Data are the mean ± SD from triplicate biological replicates measurements. Comparisons between the groups were made using an analysis of variance (ANOVA) followed by a Tukey’s test. Different letters above the error bars indicate significant differences at the 0.05 level. RPA, recombinant polymerase amplification; SD, standard deviation.

To determine the LOD, the plasmid with *N* segment of the TSWV was serially diluted by 10-fold (2.26 × 10^8^ to 2.26 × 10^1^ copies/μl) as RPA reaction templates. The results of agarose gel electrophoresis of different concentrations that were only amplified by the RPA reaction show it permitted an LOD of 2.26 × 10^3^ copies/μl (Figure 2A). Additionally, different concentrations amplified by the PCR showed an almost close result with RPA (Figure 2B). However, with the aid of the CRISPR system, the enhanced fluorescence signal showed that the limit of TSWV detection was reached at 2.26 × 10^2^ copies/μl (Figure 3). The enhancement of sensitivity of detection demonstrated that when the RPA reaction is integrated with the CRISPR/Cas13a-based detection, the system more sensitively detects the TSWV than when using normal detection methods.

### Specificity Analysis of the Tomato Spotted Wilt Virus Clustered Regularly Interspaced Short Palindromic Repeats/Clustered Regularly Interspaced Short Palindromic Repeat-Associated 13a-Based Detection

**FIGURE 4:**
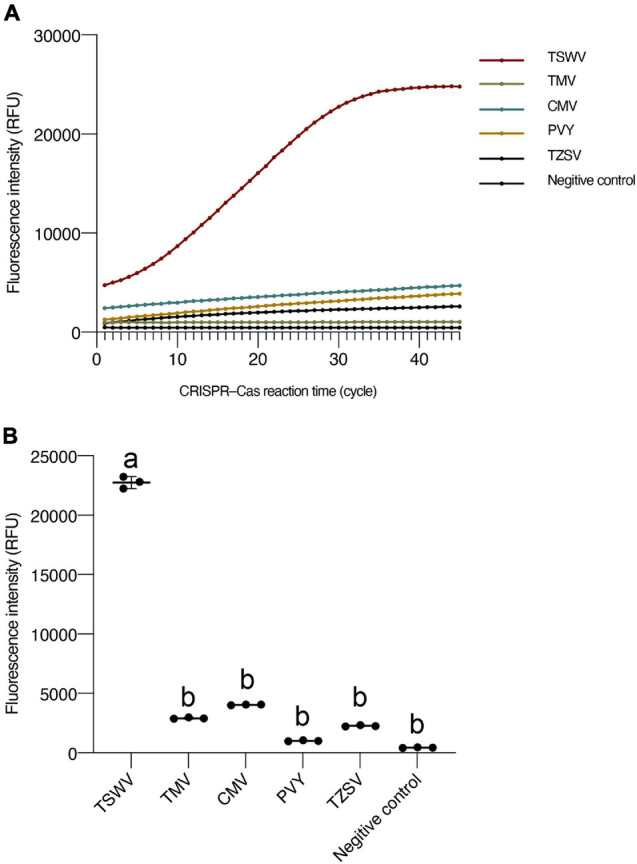
The specificity of CRISPR/Cas13a-based detection for the TSWV. **(A)** Fluorescence intensity for different viruses. **(B)** Intensity of fluorescence signals generated after 15 min (30 kinetics cycles) of CRISPR/Cas13a-based detection for different viruses. Negative control has RNase-free water as input instead of RPA production. Data are the mean ± SD from triplicate biological replicates measurements. Comparisons between groups were performed using an analysis of variance (ANOVA) followed by a Tukey’s test. Different letters above the error bars indicate significant differences at the 0.05 level. RPA, recombinant polymerase amplification; SD, standard deviation; TSWV, tomato spotted wilt virus.

To confirm the specificity of the TSWV CRISPR/Cas13a-based detection, the control tomato plants had been infected by TZSV, TMV, CMV, and PVY, respectively. The results showed that other viruses, such as the TZSV, TMV, CMV, and PVY, displayed almost unchanged fluorescence intensity compared with the TSWV sample, and that after only 15 min (30 kinetics cycle), the fluorescence intensity of the TSWV differed significantly from that of the other viruses (Figure 4). Thus, the TSWV CRISPR/Cas13a-based detection can be used to specifically detect the TSWV.

### Detection of Samples Using Clustered Regularly Interspaced Short Palindromic Repeats/Clustered Regularly Interspaced Short Palindromic Repeat-Associated 13a-Based Detection in plants and *Frankliniella occidentalis*

**FIGURE 5:**
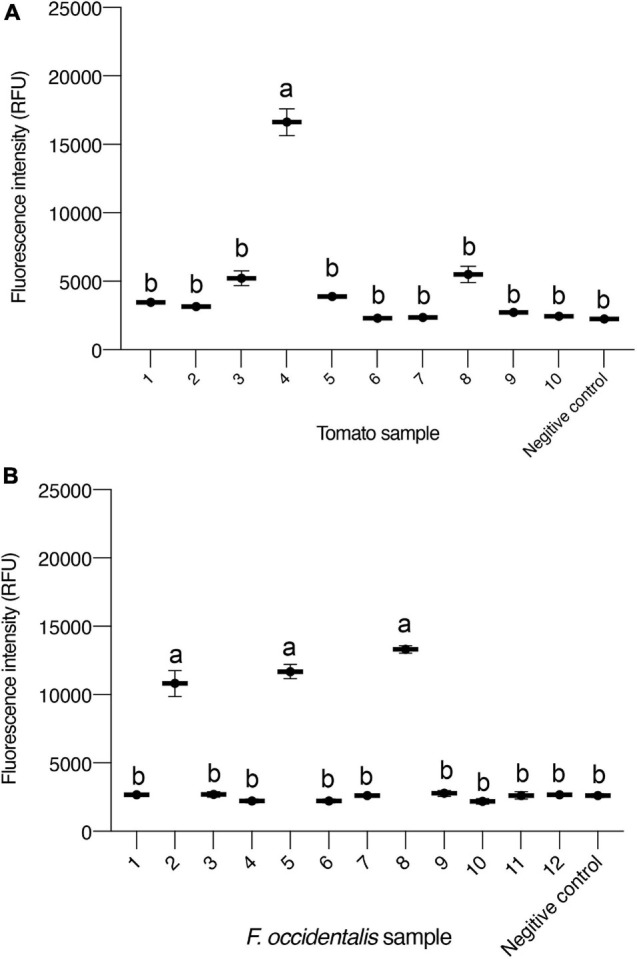
Detection of samples using CRISPR/Cas13a-based detection in plants and *Frankliniella occidentalis*. **(A)** Intensity of fluorescence signals generated after 15 min (30 kinetics cycles) of the CRISPR/Cas13a-based detection of 10 tomato samples. The negative control has healthy tomato as input. **(B)** Intensity of fluorescence signals generated after 15 min (30 kinetics cycles) of CRISPR/Cas13a-based detection of 12 *F. occidentalis* samples. Data are the mean ± SD. from triplicate biological replicates measurements. Negative control has healthy *F. occidentalis* as an input. Comparisons between groups were made using an analysis of variance (ANOVA) followed by a Tukey’s test. Different letters above the error bars indicate significant differences at the 0.05 level.

Based on the ideal result of testing sensitivity and specificity analysis of the TSWV CRISPR/Cas13a-based detection, we applied the method to detect actual samples that included tomato and *F. occidentalis*. Moreover, an RT-PCR assay was used to test the accuracy of CRISPR/Cas13a-based detection, and all the samples were tested by RT-PCR with RPA primers. The fluorescence intensity changes were monitored over the course of 45 kinetic cycles, and the intensity of fluorescence of each sample was detectable at 30 kinetic cycles. A total of 40 tomato samples and 30 thrips samples were tested (Supplementary Table 5). Among the tested samples, the RT-PCR and Cas13-based test results of tomatoes showing typical symptoms of the TSWV were the same, and 20 tomatoes showing obvious symptoms were all positive. Among the 20 tomatoes with typical symptoms and no obvious symptoms around the tomatoes, six showed positive, and the results obtained by the two detection methods were the same. We display the Cas13 test results (Figure 5A) and RT-PCR test results (Supplementary Figure 2A) of 10 tomato samples with no obvious symptoms. The result showed that the positive tomato sampled can generate significant fluorescence signal changes after 15 min (30 kinetics cycle).

*Frankliniella occidentalis* is the main transmission vector of the TSWV, a relatively effective control method is to reduce the biological population of thrips that play a very important role in quarantine and study the thrips as virus vectors. Of the tested thrips, seven showed positive results, and the results obtained by both methods were consistent. We display the Cas13 test results (Figure 5B) and RT-PCR test results (Supplementary Figure 2B) of the 12 tested thrips samples. Regardless of whether it is a tomato sample or a thrips sample, the results obtained by the CRISPR/Cas13-based detection method and the conventional virus detection method are basically the same, indicating that the method we have established has a certain usability in the detection of the TSWV and has the potential to be a kind of new TSWV detection method.

## Discussion

It is difficult to treat plants in the field when they become infected with a viral pathogen. The primary effective way to control the outbreak of the virus is by planting genetically resistant crops, planting disease-free plants, and controlling the number of vector insects during the early planting stage or at the beginning of the disease (Oliver and Whitfield, 2016). Therefore, quick, sensitive, and accurate detection assays are needed to establish a quarantine. The Cas13 protein-based detection method can be established within 1 week, with a sensitivity of >95%, and a specificity of >99%. It quickly reacts, and fluorescence results can be provided within an hour. The conditions of reaction are mild, and the reaction can be conducted at room temperature (25°C). The cost and price required to obtain the Cas13 protein is relatively low, such as USD $0.05 per test, when a mature cas13 detection method is used (Gao et al., 2021). Now, we have successfully developed a CRISPR/Cas13a-based detection platform to test for the TSWV. This method allows the tester to obtain the results of the tests 45 min after obtaining the plant cDNA. The process of TSWV detection does not require strict temperature conditions, which greatly improves the sample detection time of the assay. The conventional methods for detecting plant viruses, such as RT-PCR, require at least 2 h to obtain the detection results after obtaining the necessary cDNA sample. With the help of isothermal amplification technology, the CRISPR/Cas13a system specifically recognizes the target RNA and cleavage activity of the ssRNA probe by the Cas13a protein, combining the specific and sensitive characteristics of nucleic acid-based detection with the rapidity of antigen–antibody detection. The CRISPR/Cas13a-based dynamic detection range of TSWV was 2.26 × 10^8^ to 2.26 × 10^2^ copies/μl. This means that the presence of the TSWV can be detected during the early stage of viral infection in which the plant samples did not show obvious symptoms typical of the TSWV disease.

CRISPR-based detection methods have been well established (Xiong et al., 2020; Safari et al., 2021). In terms of reaction conditions and time, the use of RPA as an isothermal amplification reaction is more suitable to rapidly detect amplification conditions than the PCR reaction (Babu et al., 2018). As an amplification step in CRISPR detection, LAMP combined with a colorimetric reaction can also be used to visualize the results (Dao Thi et al., 2020; Joung et al., 2020; Wang et al., 2021). The detection assays to visualize the results of CRISPR-based assay system were developed by combining a lateral flow chromatographic assay with colloidal gold nanoparticles as the labeling material (Chang et al., 2020; Patchsung et al., 2020; Yuan et al., 2020; Xiong et al., 2021), and the optimization of the SHERLOCK assay enables the use of gold nanoparticles (AuNPs) to visually determine the results of the sample by a color change in the test tube (Zhang et al., 2021). The other CRISPR-type systems, Cas12, were also developed to serve as assays. The DNase activity of Cas12 is stimulated by the target DNA, and its collateral cleavage activity on the ssDNA probe reduces the risk of RNA being more vulnerable to external environmental influences in the Cas13a detection system to some extent (Aman et al., 2020; Mukama et al., 2020; Vangah et al., 2020). The Cas12 thermophilic mutant that was developed can withstand the heating conditions of the amplification reaction, so that all amplification and CRISPR-based detection reagents can be simultaneously mixed in one vessel (Ali et al., 2020). The assay that we have established still requires improvement. In future research, optimization of the TSWV assay should be conducted to obtain crude extracts of the test samples, with the goal of visualizing the results to increase the user friendliness and the components assay system to achieve mass preparation and preservation for use in resource-poor areas. Although CRISPR diagnostic technology reduces the requirement for expensive equipment and severe conditions, some of the special reagents in this system are expensive and proprietary. However, CRISPR/Cas-based diagnostics, with its advantages of stability, sensitivity, and fixed components, provides the possibility of rapid virus detection in areas with limited resources that require large number of tests (Kostyusheva et al., 2021).

## Conclusion

We developed a quick, sensitive diagnostic assay of the TSWV based on the CRISPR/Cas13a-based detection system. With only three steps, we can detect TSWV from the cDNA of infected plants and transmission vector with high sensitivity and specificity that does not require the use of time consuming and expensive equipment. The use of this method in both the laboratory and actual production process provides a new way of detecting viruses. This detection method is expected to be widely used in production and will become one of the standardized alternative methods to detect viruses.

## Data Availability Statement

The raw data supporting the conclusions of this article will be made available by the authors, without undue reservation.

## Author Contributions

FW and YJ conceived and designed the experiments. WZ and YJ wrote this manuscript and prepared the original draft. CD analyzed the data. LS and YL contributed to the reagents, materials, and analysis tools. YY, KH, and BL provided the detection samples in this study. All authors have read and agreed to the published version of the manuscript.

## Conflict of Interest

YY is employed by Country Yunnan Province Company of China Tobacco Corporation. KH is employed by Honghe City Company of Yunnan Tobacco Company. BL is employed by Sichuan Province Company of China Tobacco Corporation. The remaining authors declare that the research was conducted in the absence of any commercial or financial relationships that could be construed as a potential conflict of interest.

## Publisher’s Note

All claims expressed in this article are solely those of the authors and do not necessarily represent those of their affiliated organizations, or those of the publisher, the editors and the reviewers. Any product that may be evaluated in this article, or claim that may be made by its manufacturer, is not guaranteed or endorsed by the publisher.
