# Supplementary material for: Rapid Detection of Tomato Spotted Wilt Virus With Cas13a in Tomato and Frankliniella occidentalis
Source: Front Microbiol. 2021 Oct 20;12:745173. doi: 10.3389/fmicb.2021.745173 (PMC8564384; doi:10.3389/fmicb.2021.745173)
Supplement: Supplementary file 1 [file Data_Sheet_1.pdf]

Supplementary Material

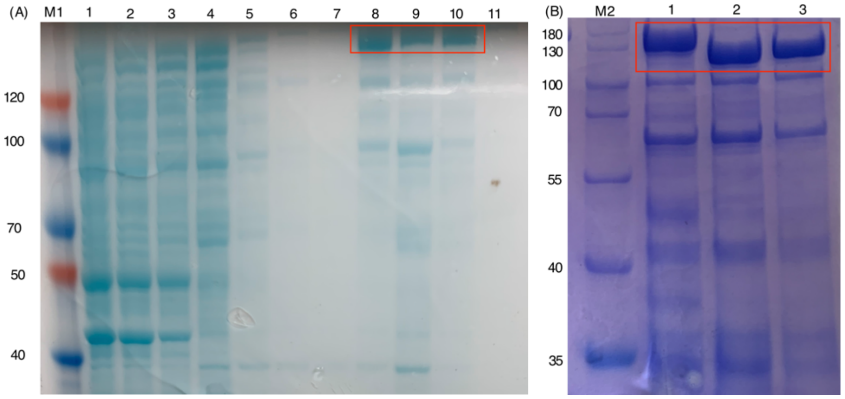

Supplementary Figure 1. Coomassie blue staining of SDS-PAGE analysis of purified LwCas13a protein. (A): His6–SUMO–LwaCas13a expression was induced by 0.5 mM IPTG at 16 °C for 18h. M1: Protein marker 120 KDa. 1-3. The first to the third flow of supernatant through the His-tag purification resin. 4-7. The first to the fourth nondenatured washing liquid collected. 8-11. His6–SUMO–LwaCas13a in the first to the fourth nondenatured elution buffer. (B): LwaCas13a was digested with SUMO protease. M2: Protein marker 180 KDa. 1. His6–SUMO–LwaCas13a in the first nondenatured elution buffer. 2-3. His6–SUMO–LwaCas13a in the first to the second nondenatured washing buffer was digested by SUMO protease at 4 °C overnight.

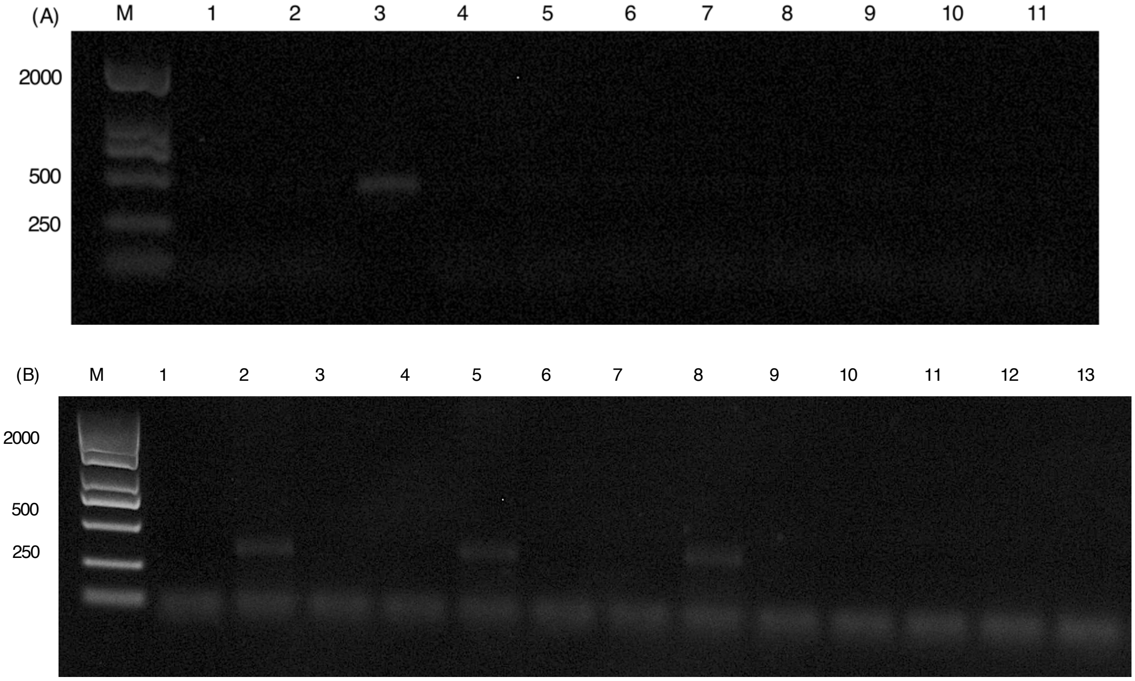

Supplementary Figure 2. The polyacrylamide gel electrophoresis result of tomato and *F. occidentalis* samples. (A) The gel electrophoresis result of RT-PCR detection of tomato samples. 1-10: tomato samples. 11. Negative control. (B) The gel electrophoresis result of RT-PCR detection of

F. occidentalis samples. 1-12: *F. occidentalis* samples. 13. Negative control

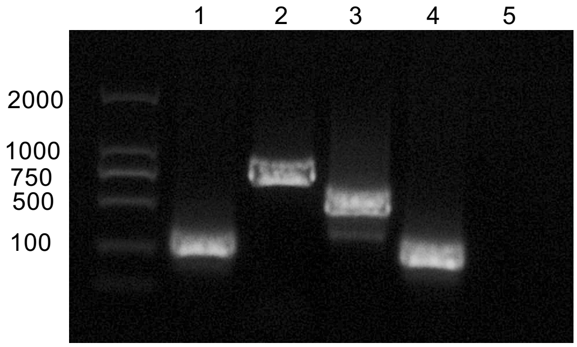

Supplementary Figure 3. The polyacrylamide gel electrophoresis result of viruses in the specific detection. 1.TMV. 2. CMV. 3.PVY. 4.TZSV. 5. Negative control has RNase-free water as an input.

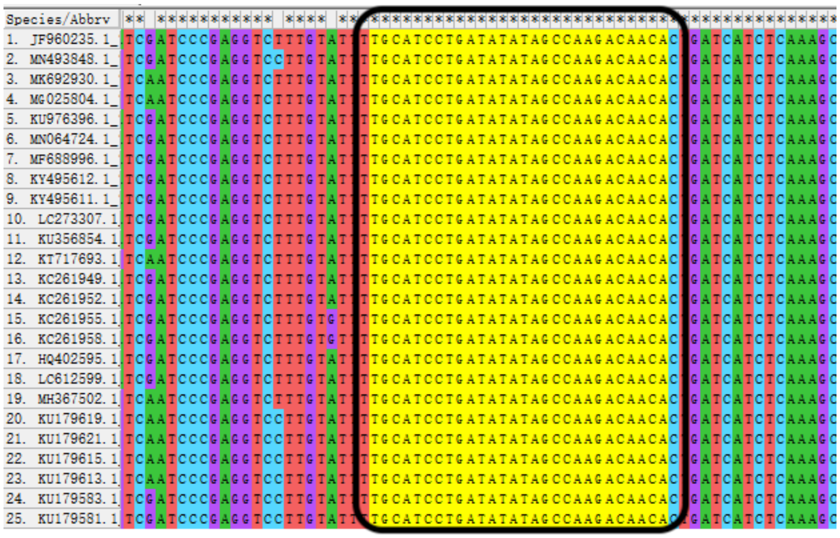

Supplementary Figure 4. The sequences of 25 TSWV isolates were downloaded from NCBI and compared by MEGAX, and the conserved sequence was selected as the target sequence of crRNA.

Supplementary Table 1 The specific primers of TSWV.

Target	Forward primer sequence	Reverse primer sequence
TSWV N	GGGTCAGGCTTGTTGAGGAAAC	TTCCCTAAGGCTTCCCTGGTG

Supplementary Table 2 RPA primer used in this study.

Target	Forward primer sequence	Forward primer sequence (with T7 RNA promoter)	Reverse primer sequence

TSWV N	ACCCTCTGATTCAAGCCTATGGATTACCTCTTG	GAAATTAATACGACTCACTATAGGGACCCTCTGATTCAAGCCTATGGATTACCTCTTG	GCACAGTGCAAACTTTCCCTAAGGCTTCCCTAG

The T7 promoter sequence was appended to 5’ end of RPA forward primer. The T7 promoter is underlined

Supplementary Table 3 crRNA sequence used in this study

Name	Complete crRNA sequence	Spacer sequence	Direct sequence
TSWV N crRNA	GAUUUAGACUACCCCAAAAACGAAGGGGACUAAAACUGCAUCCUGAUAUAUAGCCAAGACAACA	UGCAUCCUGAUAUAUAGCCAAGACAACA	GAUUUAGACUACCCCAAAAACGAAGGGGACUAAAAC

Supplementary Table 4 Annealing primer used in this study

Name	Complete sequence
crRNA-TSWV-N primer	TGTTGTCTTGGCTATATATCAGGATGCAGTTTTAGTCCCCTTCGTTTTTGGGGTAGTCTAAATCCCCTATAGTGAGTCGTATTA
T7	TAATACGACTCACTATAGGG

Supplementary Table 5 Test results of thrips and tomato samples

	RT-PCR		CRISPR/Cas13a-based Assay	
	Positive	Negative	Positive	Negative
Symptomatic tomato	20	0	20	0
Asymptomatic tomato	6	14	6	14
thrips	7	23	7	23
